# *Cases Journal*: The pitfalls to keep in mind

**DOI:** 10.1186/1757-1626-1-14

**Published:** 2008-06-04

**Authors:** K M Venkat Narayan

**Affiliations:** 1 Ruth and O.C. Hubert Professor of Global Health & Epidemiology, Emory University, Atlanta, USA

*"Perhaps the cause of our contemporary pessimism is our tendency to view history as a turbulent stream of conflicts – between individuals in economic life, between groups in politics, between creeds in religion, between states in war. This is the more dramatic side of history; it captures the eye of the historian and the interest of the reader. But if we turn from that Mississippi of strife, hot with hate and dark with blood, to look upon the banks of the stream, we find quieter but more inspiring scenes: women rearing children, men building homes, peasants drawing food from the soil, artisans making the conveniences of life, statesmen sometimes organizing peace instead of war, teachers forming savages into citizens, musicians taming our hearts with harmony and rhythm, scientists patiently accumulating knowledge, philosophers groping for truth, saints suggesting the wisdom of love. History has been too often a picture of the bloody stream. The history of civilization is a record of what happened on the banks." *– Will Durant [1]

This wonderful prose from the American philosopher, historian and writer Will Durant provides deep insight into two aspects of the psychology of humans and human societies. First, we are prone to form impressions and beliefs based on what we see, hear, experience, and feel. As an epidemiologist, I will call this the "numerator" phenomenon. Second, the news and events that make it to this numerator are often a biased selection favoring those items that are instantly dramatic, attention grabbing, or fear provoking. This is the "publication bias", which influences the media to report bad news in preference over good news and in scientific literature tends to overwhelmingly favor "positive" studies in preference to "negative" ones. [2,3]

By launching "*Cases Journal*", Richard Smith and colleagues will make an important contribution, that of providing an avenue for case reports from patients, doctors, nurses, relatives, anybody. Such a library of case reports is important, and would chronicle "the banks of the stream" in Will Durant's quote. [1] As Smith says in his inaugural editorial: "Our radical contention – which is perhaps not so radical to medical teachers – is that every case is important." [4]

While that is unquestionably true, it is important to keep the role of case reporting in perspective and also to take proactive steps to avoid some of its pitfalls. For one thing, cases will only tell us about the numerator, and only a highly select part of it. Without knowing the denominator from which the cases arise, we will never know whether the problem is truly big or small, static or growing. Continuous caution will be needed to keep cases in perspective, in the absence of denominator information, and to not allow the emotive forces behind case reporting to singly or dominantly influence policy. The potential danger of over-reacting and moving to public health policy action based on case reports alone will need to be tempered with the healthy restraint of denominator thinking. *Cases Journal *should serve more as a trigger for further thoughtful investigation or analysis and may also offer contextual information from the points of views of patients, their relatives, and the general public.

Secondly, the problem of publication bias from case reporting should always be in mind. Physicians tend to routinely "rule in" or "rule out" severe conditions, and once this is done, may get busy with other patients and duties and lose interest in pursuing a diagnosis. [5] This could result in physicians largely reporting only diagnosed severe conditions or those with atypical presentations or those that are dramatic, thus presenting a biased, incomplete and dramatic picture of the problem. Furthermore, conditions that are non-severe or difficult to diagnose may not be adequately reported by physicians. On the other hand, these conditions may be of great importance to the patients, who often want a definitive answer to their problems and can get frustrated when they don't have one. It is possible that the publication bias from patient reported cases may tend toward conditions that are hard to diagnose, label, or less severe, but are nevertheless troublesome to the patients or their families. One could argue that by opening up a channel for patients, their families, and members of the public to report cases, the new journal may provide a broader picture than case reporting simply by physicians. Nevertheless, it will be important to proactively encourage reporting of all types of cases – diagnosed or undiagnosed, mild or severe and to actively widen the net of reporting by reaching out to those who may not normally read journals. The editors of *Cases Journal *are, indeed, urging people to submit all cases [6]; they don't have to be clearly diagnosed, and they are keen to reflect the messiness and uncertainty of real life.

*Cases Journal *is a bold experiment in health journalism. It can potentially help democratize the reporting of health and disease by widening the definition of cases to include those that matter not just to physicians but also to patients, their families, and to the community at large. In the cyberage, empowerment of the public in health matters is both necessary and inevitable, and a holistic view of defining the health problems incorporating the views of the patients, their families, and the public can only be good for effective public health. *Cases Journal *can take us toward these ends. But keeping the pitfalls of case reports in the forefront of our consciousness will serve us well. As long as the editors of *Cases Journal *constantly remind the readers of the pitfalls, they will have served the cause of health and science well.
